# Towards Improved XAI-Based Epidemiological Research into the Next Potential Pandemic

**DOI:** 10.3390/life14070783

**Published:** 2024-06-21

**Authors:** Hamed Khalili, Maria A. Wimmer

**Affiliations:** Research Group E-Government, Faculty of Computer Science, University of Koblenz, D-56070 Koblenz, Germany; wimmer@uni-koblenz.de

**Keywords:** AI, SARS-CoV-2, epidemiology, interpretable machine learning, explainable machine learning, interpretable deep learning, explainable deep learning, non-pharmaceutical interventions

## Abstract

By applying AI techniques to a variety of pandemic-relevant data, artificial intelligence (AI) has substantially supported the control of the spread of the SARS-CoV-2 virus. Along with this, epidemiological machine learning studies of SARS-CoV-2 have been frequently published. While these models can be perceived as precise and policy-relevant to guide governments towards optimal containment policies, their black box nature can hamper building trust and relying confidently on the prescriptions proposed. This paper focuses on interpretable AI-based epidemiological models in the context of the recent SARS-CoV-2 pandemic. We systematically review existing studies, which jointly incorporate AI, SARS-CoV-2 epidemiology, and explainable AI approaches (XAI). First, we propose a conceptual framework by synthesizing the main methodological features of the existing AI pipelines of SARS-CoV-2. Upon the proposed conceptual framework and by analyzing the selected epidemiological studies, we reflect on current research gaps in epidemiological AI toolboxes and how to fill these gaps to generate enhanced policy support in the next potential pandemic.

## 1. Introduction

The application of artificial intelligence (AI), especially machine learning and deep learning models, has been evidenced in a variety of research areas such as computer vision, robotics, epidemiology, medical imaging, etc., as one of the most powerful approaches to contain the spread of the SARS-CoV-2 pandemic [1]. While the excellence of AI models in terms of their accuracy and performances is broadly admitted, the results and prescriptions made based on these models are not always as transparent as required [2]. In other words, despite being highly accurate, AI models are not sufficiently interpretable (or explainable). Miller defines interpretability as “the degree to which a human can understand the cause of a decision” [2]. The terms explainability and interpretability are often used interchangeably. Explainability is also referred to in the literature as interpretability, intelligibility, causability, or understandability [3].

In recent research, explainable artificial intelligence (XAI) has received high attention to address interpretability. The goal of XAI is to understand and explain the corresponding processes behind the algorithms, which lead to the generated predictions of the AI models [3,4,5,6,7,8].

In AI studies of the SARS-CoV-2 pandemic, XAI remains one of the main concerns, especially with regard to medical AI systems [9], as AI-based medical diagnoses are directly linked to human lives. While the significance of XAI in healthcare systems is self-evident, the substantial role XAI may play in AI-based and data-driven generation of government policies in pandemic circumstances is a noticeable subject as well [10].

**Figure 1:**
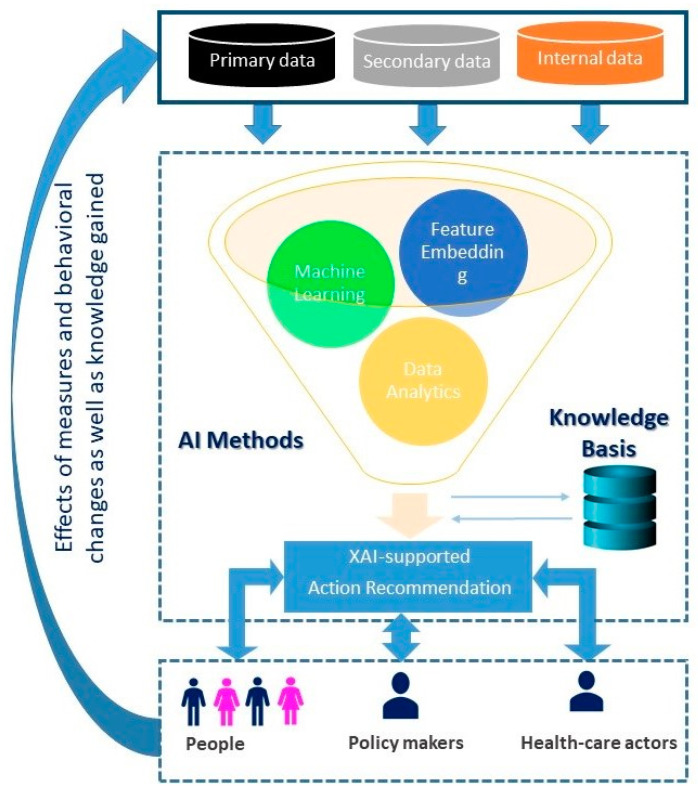
Motivation of applying XAI to influence the understandability of AI at policy level along the overall AI and COVID approach (https://covid-ai.uni-koblenz.de/, accessed on 20 June 2024).

Figure 1 shows an example of a basic AI-powered recommendation system to contain the SARS-CoV-2 pandemic, researched within the “AI and COVID project”. The system uses three types of databases: (a) Publicly available information on the global status of the pandemic and historical data on measures and their impacts (primary pandemic data), (b) region-specific information on population and demographic characteristics (secondary pandemic data), and (c) information about the current status of available healthcare personnel and resources (internal data). By developing AI-supported methods and taking into account the current knowledge base, recommendations for actions are developed and communicated to the key pandemic-relevant target groups, including political decision-makers, health authorities, and citizens. The impact of the proposed policy measures, e.g., pharmaceutical interventions (PIs) and non-pharmaceutical interventions (NPIs), behavioral changes, and knowledge gained are then stored back into the primary pandemic databases for use in the next phase of the pandemic.

XAI makes it possible to transparently present the significance and magnitude of the recommended measures for the target actors mentioned above. In particular, in the presence of XAI, the policy level can see the evidence of the results achieved at the level of AI development and trust the proposed policies.

In addition, system developers can also benefit from the use of XAI at the AI development level. XAI enables system developers to take a look at the internal workings of AI-based algorithms and helps them eliminate potential pitfalls (e.g., misunderstandings of semantics and syntax errors) that arise at the AI development level and correct the corresponding errors [11].

Identifying the main methodological gaps in the AI pipelines of epidemiology remains important to develop enhanced epidemiological XAI toolboxes for the next potential epidemics. In order to develop a comprehensive understanding, our objective in this paper is to systematically review the existing literature in the field and to figure out the main research gaps on XAI and AI-based epidemiological pipelines of the recent SARS-CoV-2 pandemic. The insights obtained from our review serve as a guide for expanding the methodological toolbox in AI-based epidemiology. The following research questions (RQs) drive this study:

RQ 1: What is the current state of research on XAI applied to the recent pandemic, and what research gaps exist?

RQ 2: What would be a suitable conceptual framework to systematically analyze the main methodological features of interpretable machine learning pipelines on SARS-CoV-2 data?

RQ 3: What further research is required to boost the development of explainable AI models of epidemiology?

To answer these RQs, the study conducts a systematic literature review by adapting the Preferred Reporting Items for Systematic Reviews and Meta-Analysis (PRISMA) approach [12,13].

The remainder of the paper is as follows. Section 2 details the methodology for the systematic literature reviews in the subsequent Section 3 and Section 4. In Section 3, a literature analysis is conducted with the focus of conceptualizing a decent framework of interpretable machine learning pipelines in the context of SARS-CoV-2. Based on the conceptual framework, in Section 4, we focus on the epidemiological AI approaches of SARS-CoV-2 and analyze the set of selected epidemiological papers. Section 5 concludes the paper with a discussion and reflection of future research needs derived from the gaps identified.

## 2. Research Methodology

Our study applies the Preferred Reporting Items for Systematic Reviews and Meta-Analyses (PRISMA) approach for literature identification [12]. The process employs four sequential phases: Identification, screening, eligibility, and inclusion. The identification phase is carried out by filtering titles and content in the Google Scholar database. Thereby, we opted for broad coverage by using the search term “COVID” in the title as well as one of the semantically exchangeable search terms “interpretable machine learning”, “interpretable deep learning”, “explainable machine learning” and “explainable deep learning” in the body text (accessed on 10 January 2024). We opted for setting a generally defined search term in the database without primarily specifying any keywords with relation to the “epidemiological” target of our review. This was conducted to minimize the risk of bias in the selection of the final included studies in favor of specified words such as epidemiology, government policy, non-pharmaceutical interventions, etc. The search of the aforementioned four search combinations resulted in a total of 1503 studies. As among the collected papers, 218 studies were duplicates due to coexistence in more than one of the four aforementioned search combinations, the duplicated papers were filtered, and we moved on with 1285 papers in the screening phase.

In the screening phase, an Excel sheet was used to mark each paper with regard to the specific research area (with respect to the methods and results) it belongs to. For the development of the reference framework for interpretable machine learning pipelines on SARS-CoV-2 data, the 1285 papers were the basis. We particularly scanned the studies for the main methods of data processing and ML processing pipelines. This resulted in 325 papers that deal with different methodological features. Through tabular evaluation, we analyzed these papers to synthesize the information with regard to the six steps for our conceptual framework. A tabular representation of the papers is presented in the paper’s Supplementary Material. The results of the synthesis are described in Section 3 along with the conceptualized reference framework of the pipeline. This reference framework was applied to identify both the potentials and limitations of the narrowed scope of epidemiological AI research in the SARS-CoV-2 context elaborated in Section 4.

To narrow the vast amount of literature for Section 4, more than seven research areas were identified to group the studies. A subset of identified papers included 328 papers related to X-ray and CT imaging classification methods. 196 papers addressed SARS-CoV-2 diagnosis approaches based on clinical markers (i.e., blood tests, patient symptoms etc.). 62 papers tackled psychology and language understanding. Further 611 papers were grouped into the miscellaneous area. While, within this miscellaneous group, we identified 188 articles that conducted reviews of literature in the scope of AI research and SARS-CoV-2 epidemiology from different perspectives, none of these studies have focused on the same objective as our study, i.e., jointly incorporating AI, SARS-CoV-2 epidemiology, and XAI. We therefore did not include these papers in our further consideration for the latter analysis of epidemiological AI research with XAI. 88 studies were assigned to the epidemiological category to be further considered in our targeted group of relevant studies in Section 4.

**Figure 2:**
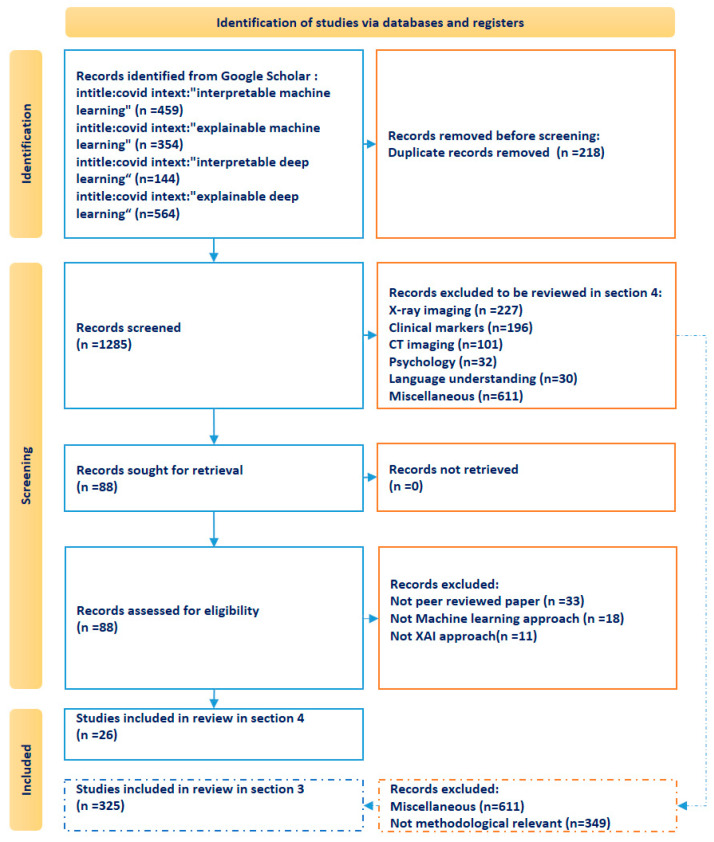
Study search and selection process for literature on epidemiological AI research based on the PRISMA template [13]. The dashed lines and dashed squares are added to elucidate the selection process of included studies reviewed in Section 3.

Next, we further screened the eligibility of the 88 epidemiological studies as the main study target with the following three inclusion rules: (a) Being a peer-reviewed journal paper, (b) using at least one ML model rather than statistical regression models in the paper, and (c) that the paper applies XAI in the pipeline. By stepwise filtering based on the three inclusion rules, we finally included 26 papers, which are further categorized and explored in more detail in Section 4 of this paper. Figure 2 presents the flowchart for selecting the papers for the epidemiological AI research in Section 4 based on the PRISMA 2020 flow diagram for new systematic reviews [12,13].

## 3. Literature Review on XAI Pipelines of SARS-CoV-2 and Reference Framework for the Subsequent Epidemiological Study

**Figure 3:**
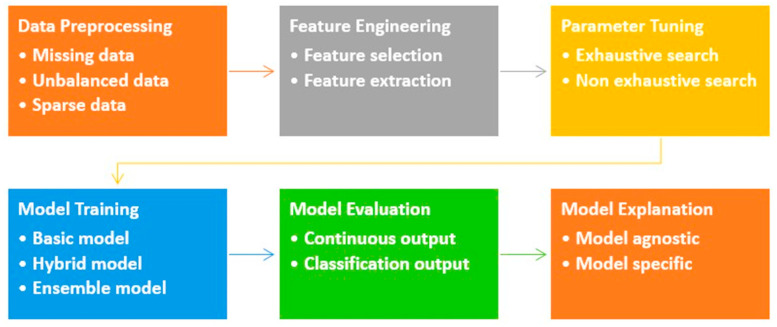
A conceptual reference framework of ML pipelines in the context of SARS-CoV-2.

In this section, we review the existing literature with regard to XAI applied in the identified SARS-CoV-2 literature. The objective is to conceptualize a reference framework for studying XAI pipelines of SARS-CoV-2 for synthesizing the main methodological features in Section 4. The proposed reference framework (Figure 3) entails the main steps of Severn et al.’s approach, i.e., process data, build prediction models, and explain prediction model sub-pipelines for explainable machine learning models [14]. However, we followed a more fine-grained and adjusted set of steps as shown in Figure 3: Data preprocessing, feature engineering, parameter tuning, model training, model evaluation, and model explanation (XAI). This more detailed pipeline is extracted from the literature analysis and is described below along the individual steps.

### 3.1. Data Preprocessing

The Data Preprocessing step handles missing, unbalanced and sparse data.

#### 3.1.1. Missing Data

Missing data can cause concerns not only to the model’s precision but, furthermore, to the interpretation of the achieved output. While missing data values are inherently handled by some techniques, e.g., gradient-boosting predictors [15], these remain a basic problem on top of the pipeline in numerous studies. The lack of enough data hampers the prediction accuracy of SARS-CoV-2 cases, while having larger datasets can lead to improved results [16]. As the amount of data increases, the epistemic uncertainty related to the model decreases [17]. In contrast, the problem of lacking data is amplified when facing problems with a large number of inputs. Docquier et al. show how the inclusion of extra parameters (to incorporate day-specific effects—i.e., 366 day-specific time dummies) in their models deteriorates the predictive power of the ML model. The authors conclude that, given a dataset, the gains from adding information are indeed outbalanced by the costs linked to the inflated dimensionality of the AI computation problem [18]. Hinns et al. study XAI generated by various predictors on a dataset and show how inconsistent model interpretations emerge among a set of random data sub-sets when using little data, and that by increasing the size of the data, interpretations of the random data subsets converge towards each other [19]. Andonov, Ulm, and Graessner show how a sudden shift in the input data impacts the performances of AI models as well as the explanation of the models [20].

To combat missing data, in the context of SARS-CoV-2 studies, the k-nearest-neighbor algorithm (KNN) is frequently used [21] to impute the missing values in the dataset. By trying to compare an unlabeled data point to the training dataset, the KNN finds the K most related data points. Thereby, a metric that measures distance, such as Euclidean or Manhattan distance, is utilized to determine proximity. This technique then assigns the given data point to the most familiar class [22]. Another possible imputation approach is the usage of generative adversarial neural networks (GANN), which learn to generate “missing” data with the same distribution as the training set. This is performed by training a “generative” network, which generates possible imputed values and proposes them to a “discriminative” network, which is trained to accept only those generated values that properly fill the missing ones according to the underlying data distribution [23].

#### 3.1.2. Unbalanced Data

Unbalanced data can lead the model’s performance to be biased in favor of the classes or ranges of outputs, which are overrepresented. The unbalanced data is often handled by the Synthetic Minority Oversampling Technique algorithm (SMOTE) [24]. SMOTE works based on identifying the k-nearest neighbors’ principle and deploys the principles of interpolation [25]. It creates synthetic data that is close to the minority class to oversample the minority class in the feature space [26,27]. To handle the problem of unbalanced data, other alternatives exist, such as data partitioning (i.e., utilizing dichotomous variables). Discretizing the variable spaces will not necessarily worsen the model performance in all circumstances to a large extent. For instance, Wendland et al. show that models using only dichotomous features perform only slightly worse than models based on a complex combination of numerical input values [28]. Another way of managing unbalanced data is during the training step. In [29], during the fitting procedure, the unbalancing issue is tackled by penalizing the misclassification of the minority class with a multiplicative factor inversely proportional to the class frequencies. Hu et al. propose a novel self-adaptive auxiliary loss to help the training with imbalanced data [30]. The self-adaptive factor reflects the feature distribution and emphasizes the minority class. Also, other data imputation methods are used in the SARS-CoV-2 literature based on decision trees, e.g., isolation forest [31], miss forest [32], and random forest [33]. The range of possible data imputation techniques is not restricted to those frequently used. For example, predictive mean matching to impute numeric features, logistic regression to impute binary variables, and Bayesian polytomous regression to impute factor features are used in [34]. In addition, Abbasimehr, Paki, and Bahrini present a time series augmentation technique to create new time series with the same temporal dependencies that exist in the original time series data [35].

#### 3.1.3. Sparse Data

To resolve the generic problem of sparse training data, generative networks (GANs) are applied in studies to generate ample synthetic training data [36,37,38]. With limited data in the SARS-CoV-2 context, synthetic data is generated using the auto encoder (AE) methods [39,40,41]. AEs belong to the realm of unsupervised learning, as they do not need labeled data for their training. The process consists of providing labeled sample data to the encoder, which captures the distribution of the deep feature, and the decoder, which generates data from the deep feature by decompressing the latent space.

### 3.2. Feature Engineering

Feature engineering generally covers feature extraction and feature selection [42]. Whereas feature extraction creates new features, feature selection is about selecting a subset of the original feature set [43]. For feature extraction, two types of methods are distinguished: pre-trained feature extracting and reduced dimensional feature extracting.

#### 3.2.1. Pre-Trained Feature Extracting

As most ML models require inputs in the form of numerical vectors, some feature extraction techniques aim at translating features such as vocabulary [44], images [45], or parts of speech into numerical representations. This is performed in most image recognition studies [46] as well as natural language processing studies [44] by means of pre-trained deep learning models. The pre-trained model acts as an early feature extractor, usually followed by a fine-tuning step [47]. Subsequently, a downstream classification step is executed in many cases [48].

The upstream part of an ML pipeline can comprise the translation of, e.g., text and image in both directions to extract desired features. For example, Shang et al. utilize a text-guided visual feature generator to generate visual features from the news text as well as an image-guided textual feature decoder to generate the corresponding textual features from the news image [49].

#### 3.2.2. Reduced Dimensional Feature Extraction

Feature extraction techniques can also aim at learning the reduced structure of the data by finding a low-dimensional embedding representation that preserves the essential structure of the data. For this, a variety of algorithms are applied in the context of the SARS-CoV-2 literature, as summarized in Table 1.

Docquier, Golenvaux, and Nijssen use a PCA analysis to reduce the dimensionality of the origin- and destination-specific containment measures, extract the first two components of the PCA, and propose that the first PCA component can be interpreted as an average index of the stringency of containment measures, and the second component captures testing and tracing policies [18]. Trajanoska, Trajanov, and Eftimov cluster countries with similarly balanced diets using SOM. In addition to presenting the SOM clusters, the authors present an explainable decision map corresponding to the SOM clusters, with squares representing the most dominant feature leading to the decision to cluster the countries [61].

Beside the previously mentioned methods, knowledge graph embedding techniques to encode the entities and relations in a knowledge graph as dense and low-dimensional vector representations are utilized in the literature of SARS-CoV-2 [62,63]. In addition, functional data analysis following the principle of “breaking up the whole into pieces” of big data analysis to transfer discrete and high-frequency sequences of data to continuous smooth functions, treating the whole functions as a single entity with an internal unified structure, is used in the literature [64].

While the above-mentioned techniques for reducing the number of variables can eliminate redundant and irrelevant features, de Paiva, Pereira, and de Andrade argue that it is not always clear whether these methods result in improvements in the predictive power of ML models [65]. Furthermore, as these methods project the features to a new dimension and the features in the new dimension become mixed features, these new features might not necessarily provide a strong explanatory basis [66]. Despite that, some of the aforementioned studies have provided XAI along with the corresponding AI algorithms. For example, [58] constructs discriminative decision rules that identify and differentiate the clusters, forming the explanations of subgroups. Moreover, features with the strongest impact on clustering can be examined by assessing their importance to each emerging cluster through supervised machine learning models and subsequent application of XAI techniques [67].

#### 3.2.3. Feature Selection

Feature selection aims at eliminating irrelevant and redundant features. Irrelevant and redundant features not only increase the computational complexity of a model but also increase the probability of overfitting [68].

Statistical correlation analysis is the first milestone to observe if, e.g., there is a high degree of correlation between multiple independent variables. Finding high correlations between two variables, e.g., the share of the population with cancer and the share of the elderly, is conceivable. After the correlation analysis, such variables can be reduced to continue the investigation with a lower number of representing variables [69]. The factor analysis technique is an alternative statistical method that extracts the maximum common variance from all variables and puts them into a common score. This contributes to identifying latent composite variables, for example, between gross domestic product (GDP) per capita and other development metrics, such as access to electricity [70].

Various statistical methods can contribute to evaluating the association between independent variables and the dependent variables (leading to sorting the priority of influential variables and eliminating the irrelevant ones), including H-statistics [71], Pearson’s correlation analysis [72], chi-square [73], T-test [74], U-test [51], univariate logistic regression [75], etc. While statistical methods can indicate the overall interaction strength of each feature with the other features, they do not convey what the interactions look like. That is what XAI is for.

Next to the statistical approaches, the selection of the feature selection model is often based on training an ML model. This ML model could be identical to the training model at the upcoming stage of the pipeline or not. Various approaches undertake a kind of stepwise wrapping feature selection by removing (or adding) features one by one from (to) a set of features and evaluating the model error (or statistical significance of the added factor) through training the model at the upcoming stage of the pipeline (forward feature addition and backward feature elimination) [69,76].

Alternatively, the selection of the feature selection model can be carried out based on training an ML model and computing the significance of each feature through the subsequent XAI corresponding to the chosen ML model [77,78]. For example, a number of studies utilize the SHAPley value-based explanations (SHAP) concept (see Section 3.6 below on model interpretation) to undertake the task of feature selection in their pipeline [52,79,80,81].

Beside the above-mentioned categories (i.e., statistical analysis, error-based, and XAI-based feature selection methods) different evolutionary techniques are utilized in the context of SARS-CoV-2 literature for feature selection. Examples are artificial bee colony, ant colony optimization, butterfly optimization algorithm, elephant herding optimization, genetic algorithm, and particle swarm optimization [40,82,83].

An alternative kind of explainable feature selection is proposed by [84] by initializing a weighted graph to comprise features with Pearson similarity criteria for the feature similarities calculation as well as the integration of Fisher score (FS) and the node centrality to determine the score of each feature. That way, the feature selection approach considers not only feature importance but also feature similarity.

**Figure 4:**
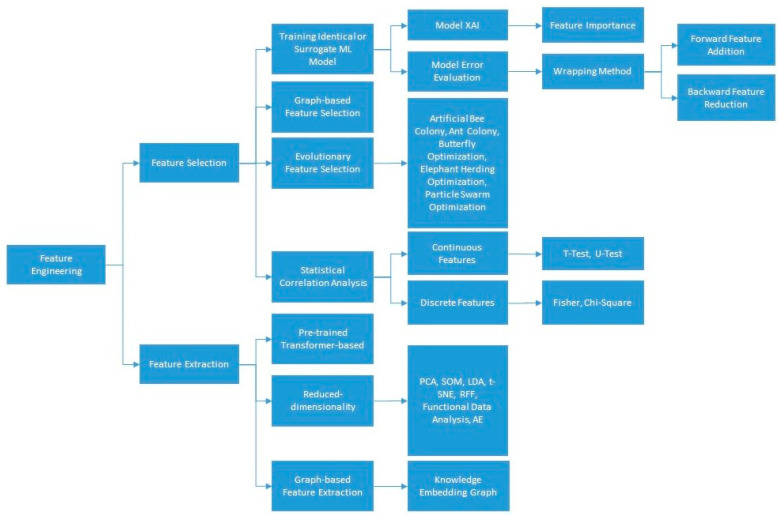
Overview of the feature engineering approaches resulted from the SARS-CoV-2 research studied in Section 3.2.

Figure 4 portrays an overview of the methods presented in Section 3.2.

### 3.3. Model Parameter Tuning

To achieve highly precise results through ML algorithms, various approaches are used to fine-tune the models’ hyper-parameters. The hyper-parameters of DL (deep learning) models, which must be tuned, consist of the number of layers, number of neurons, activation function, learning rate, etc. [72]. For example, Vernikou, Lyras, and Kanavos show that the Bert Tokenizer long short-term memory network (LSTM) model responds better with a very low learning rate [85]. The hyper-parameters to tune in decision tree-based models would comprise the maximum depths of trees and the maximum number of features used in each split. Hyper-parameter tuning is conducted in most pipelines with a grid search algorithm. The grid search algorithm tests all combinations of hyper-parameters and narrows the model parameters to the optimal ones [25,86,87].

Due to the computational costs of information processing in grid search strategies, evolutionary and swarm intelligence-based optimization algorithms are also applied [88,89]. In addition, the search for hyper-parameters is carried out frequently using a Bayesian search [90,91,92].

### 3.4. Model Training

**Figure 5:**
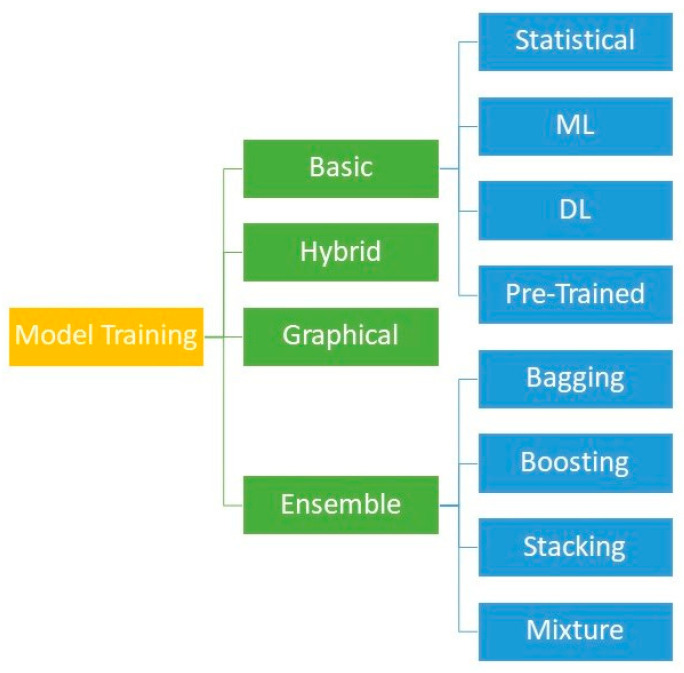
Overview of the model training approaches resulted from the SARS-CoV-2 research studied in Section 3.3.

Model training approaches are based on different categories consisting of statistical regression models, pre-trained DL (deep learning) models, ML basic models, DL basic models, graph models, ensemble models, and hybrid models. A complete list of detailed training (as well as interpretation) methods used in the identification phase of the literature in our paper is provided in the Supplementary Material to this paper. Among statistical regression models, the logistic regression (LR) model is frequently used to take on either classification or regression tasks in various studies [93,94,95]. Model training in the context of natural language processing and medical imaging is often elaborated through pre-trained models, such as Bert [44], ResNet [96], etc. Among the basic ML models, the extreme gradient boosting algorithm (XGB) is elaborated on in a variety of classification or regression tasks in the context of the SARS-CoV-2 literature. XGB itself is an ensemble model encompassing multiple weak tree-based models, which work together [97] based on the boosting approach. Boosting is a sequential ensemble method that iteratively adjusts the weight of observation as per the last model output. Long short-term memory networks (LSTM) and convolutional neural networks (CNN) are prominent examples of basic DL models applied frequently in the context of SARS-CoV-2 studies. LSTM excels at capturing time data dependencies, making it ideal for sequence prediction tasks [98]. CNN is specifically used for image classification and tasks that consider the processing of spatial dimensions of data [99]. While CNNs are primarily used for computer vision applications, they work on different time series problems, too [100]. Ensemble models incorporate a number of basic ML or DL models to achieve higher degrees of accuracy. Four main alternatives to creating ensembles, comprising bagging, boosting, stacking, and mixture of experts, are addressed in [93]. Hybrid models concatenate different combinations of ML and DL models at different model architecture levels [96,101,102]. Graph models reflect the underlying logical connection of the model components in a graphical style [103,104]. Graph neural networks (GNNs) are novel graph models that comprise input variables as graph components, e.g., nodes and edges. The graph components get updated through machine learning models, e.g., based on the feature networks of the nodes’ neighbors and the edges connecting them [105]. Figure 5 provides an overview of the methods presented in Section 3.4.

### 3.5. Model Evaluation

The evaluation of the performance of an ML or DL model depends on the model outputs. For pipelines with classification output types, the evaluation criteria are often AUC (area under receiver operating characteristic curve), precision, recall, F1-score, and accuracy [106]. The aforementioned criteria can be further elucidated based on the notions of the receiver operating characteristic (ROC) curve [107] as well as the precision and recall (PR) curve [108]. To evaluate the performance of pipelines with continuous outputs, mean absolute error (MAE), mean square error (MSE), root mean squared error (RMSE), and goodness-of-fit (R2 score) are used [90]. Furthermore, instead of evaluating the performance of a model on a single validation dataset, multiple random splits (k-fold validation) are utilized (as the performance of a model can change depending on the choice of split) [109]. For the time series ML models, a sliding window can be applied to the dataset to alter the test data that is set in a non-random (subsequent) manner from a time point to the next time point onward. Thus, if, e.g., for the first split, the test set covers 20% of the earliest data records from a certain time point on, the test set of the last split corresponds to the most recent 20% of the data records [77].

### 3.6. Model Interpretation

ML and DL models can be grouped into two categories of interpretability: Intrinsically interpretable and non-intrinsically interpretable [3]. A complete list of detailed interpretations (as well as training methods) used in the identification phase of the literature study can be found in the Supplementary Material.

#### 3.6.1. Intrinsically Interpretable Models

Statistical regression models, e.g., a logistic regression (LR) model, are examples of intrinsically interpretable models. In these models, a coefficient or odds ratio summarizes the positive or negative strength of the association between exposure and an event. Moreover, the coefficients from the LR model can be utilized to build a nomogram predicting the model outcome [110]. Regressions remain one of the simplest and most explainable models with a clear formulation. Despite this, they may not precisely accommodate the non-linear and non-monotonic patterns in the data. The literature of SARS-CoV-2 studies has proposed a range of intrinsically interpretable methods rather than statistical models. Table 2 summarizes a list of intrinsically interpretable methods identified throughout the literature analysis.

#### 3.6.2. Non-Intrinsically Interpretable Models

The non-intrinsically interpretable models are analyzed in two alternative ways in the literature: Model-agnostic approaches and model-specific approaches [3,4,5,6,7,8]. Model-agnostic approaches presume ML models as a black box and try to convey XAI based on surrogate models, either by means of employing intrinsically interpretable meta-explaining models or by means of employing perturbation mechanisms. Model-specific approaches, conversely, try to embed XAI into the specific model to observe the feature influences during the training procedure. Among model-agnostic approaches, SHAP is frequently elaborated on in a variety of model interpretation tasks in the context of the SARS-CoV-2 literature. The SHAP is a perturbation-based concept. Perturbation-based approaches aim at analyzing the importance of each input on the model outcome by systematically modifying the input of the model and observing the changes in the output. If the permutation of a specific part of the input, considerably alters the model output, then the specified part is considered to be important. SHAP computes the average marginal contribution of a feature to the output predicted by the ML model, considering all possible combinations of features [118]. The SHAP computation time increases exponentially with the number of features.

Local interpretable model-agnostic explanation (LIME) is another most frequently applied interpretation model-agnostic method that is based on perturbation and meta-modeling. LIME tunes the values of the features of a selected predicted instance and generates new samples based on the proximity to the instance being picked [119]. It then optimizes a line based on all generated samples and gives a local interpretable explanation of the instance being picked.

Likewise, a number of studies in the context of the SARS-CoV-2 literature have utilized other surrogate meta-models, which are intrinsically interpretable, to explain the logic behind decisions made by an original black-box model. Examples of such approaches include:

- using formal concept analysis (FCA) to create a set of association rules with different confidence intervals [120];
- applying a Bayesian network to visualize the effect of the potential influencers on decision making [24];
- proposing a single associated decision tree (DT) to represent a random forest (RF) model [68];
- applying the anchors method to help explain predictions by decision rules [23];
- utilizing a probabilistic graphical model (PGM-Explainer) as a simpler interpretable Bayesian network in order to interpret GNNs [121];
- applying the symbolic meta modeling approach, which integrates various simple parameterized functions to obtain a closed-form and interpretable expression for the meta model [122].

Despite a wide range of practical applications of model-agnostic models, these approaches are not ideal XAI approaches, representing the original training procedures behind the model they explain. Model-agnostic methods are indeed surrogates, which first presume an ML model as a black box, then derive their interpretations (after the model training is finished) from a different modeling perspective with priors that are not necessarily in line with the internal procedures of the original model [123]. Model-specific approaches try to fill this gap by trying to provide information regarding the actual reasoning process within the specific model through the training. Model-specific approaches are built based on incorporating weights, gradients, or attention from DL model-specific layers.

**Figure 6:**
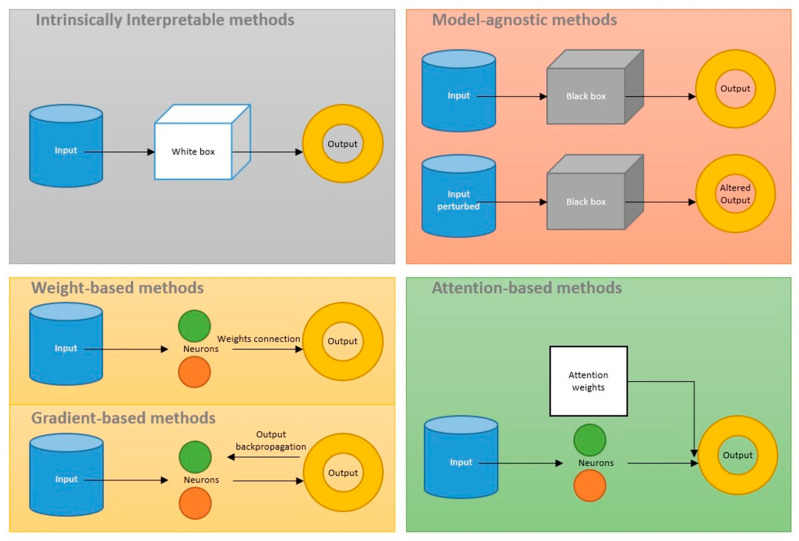
Overview of the mechanisms of XAI approaches used in the SARS-CoV-2 context: intrinsically interpretable methods, model-agnostic methods, weight-based methods, gradient-based methods, and attention-based methods.

Figure 6 visualizes the mechanisms behind the XAI approaches reviewed in this section.

Weight-based techniques utilize the product of final weights based on the connections from input neurons to the output neurons [72,124]. Gradient-based techniques back-propagate outputs onto a particular feature map (the output of one filter applied to the previous layer). Usually, the feature map is chosen to be the final convolutional layer in CNNs. Class activation map (CAM) and Grad-CAM explanation methods are frequently applied examples of weight-based and gradient-based approaches in the literature [125]. CAM uses the notion of global average pooling (GAP) and learns weights from the output of the GAP layer onto the output classes. Grad-CAM generates a localization map that shows the critical features by using gradients from the target class, which are settled in the final convolutional layer in the CNN network [125]. Integrated gradients (IG) method is another gradient-based alternative. IG methods examine the inputs of a deep learning model and their importance for the output by integrating the gradients of the output with respect to the input along an arbitrary path from the baseline to the input data point [98].

In addition, attention mechanisms have gained a lot of attention in the SARS-CoV-2 literature [126]. The attention approaches are inspired by human attention visual mechanisms, which use limited attention to quickly screen high value information from a large amount of information. This not only contributes to increase the prediction performance but is also efficient in gaining insight into information that is more critical to the model outputs instead of learning non-useful information [37,127,128].

## 4. Literature Analysis of Epidemiological AI Research

In this section, we focus on the 26 papers that fulfill the inclusion criteria of our study. A detailed tabular representation of the 26 papers (comprising research question, data, pipeline, and significant results per paper) is presented in the paper’s Supplementary Material.

The models in the papers are designed to analyze different epidemiologic aspects of SARS-CoV-2 in different geographical scopes. A subset of papers incorporated data of multiple countries, including analyzing NPIs in 176 countries [129], analyzing the evolution of cross-border movements of people during the SARS-CoV-2 in Europe [18], studying the influence of NPIs, PIs, virus variants etc. on SARS-CoV-2 spread in Europe [130], investigating the role of booster vaccine in 32 countries [131], focusing on the role of dietary imbalances in 154 countries [61], assessing the role of NPIs against SARS-CoV-2 at containing seasonal influenza transmission in 33 countries [92], forecasting confirmed cases prediction in 8 countries [102], assessing the effect of non-countermeasure factors (e.g., cultural factors) to classify countries into those more and less prone to the fast spread of SARS-CoV-2 [51], and explaining a variety of socio-temporal variables on SARS-CoV-2 prevalence and mortality at a global scale [72].

The rest of the papers, e.g., [98,127,132], mostly utilize the data of one country or region, especially from the US. The analyzed studies focus on different research objectives. Depending on the focus, they generate different explanations on the significance of different influential factors on the spread of the pandemic, such as compliance with interventions [133], population density [134], population movement and gathering [76,78,92], lock down effects [18,120], labor and unemployment effects [72,81], closure and regulation of schools [129,135], vaccination [130,131,132], spatial effects [74,136], weather conditions [127,137,138], country dietary and cultural effects [51,61], virus variants [98], and health infrastructural impacts [139].

The data preprocessing stage encompasses, in most studies, a data imputation step. In rare cases, the unbalanced data issue is handled, e.g., by SMOT in [138] and by excluding those NPIs that were used in less than 20 countries [129]. Data discretization is carried out by means of a relevant algorithm due to the necessity of discretization of feature values in Bayesian network analysis (but not for the sake of handling data unbalance) [136].

Feature extraction techniques are applied in three studies, comprising: PCA analysis in [18], SOM in [61], and K-means in [51]. The feature selection step is performed in most of the studies. It consists, e.g., of statistical analysis in [72,133], and stepwise wrapping methods based on k-fold validation [61,76,92,136,137,139].

SHAP-based feature selection methods [61,78,92] and feature selection based on alternative ML methods [51,92] are applied as well. Data preprocessing and data engineering steps are not explicit parts of the pipeline in [74]. The authors use the inherent advantage of XGB to cope with correlations between the covariates and to deal with data imputation.

Parameter tuning and model evaluation stages are in most models performed based on a grid search algorithm and by evaluation metrics as introduced in Section 3.3 and Section 3.5, respectively.

**Figure 7:**
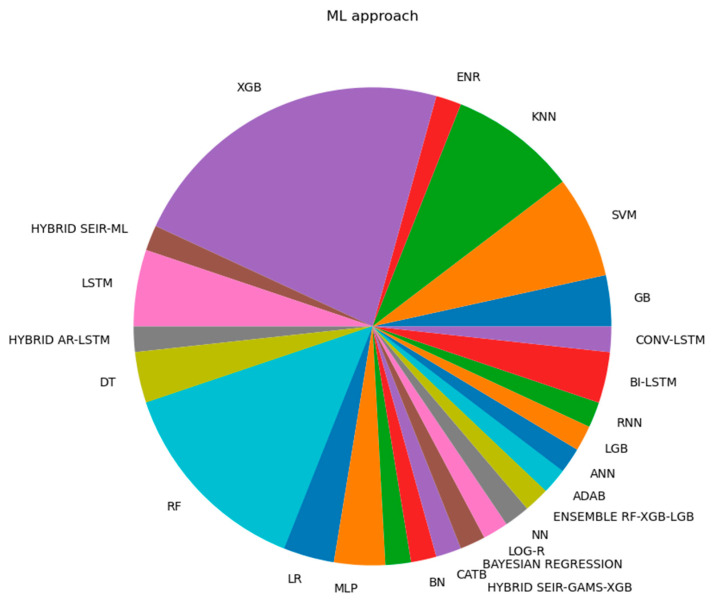
ML approaches used in the epidemiological context, and frequency of use in the studies.

**Figure 8:**
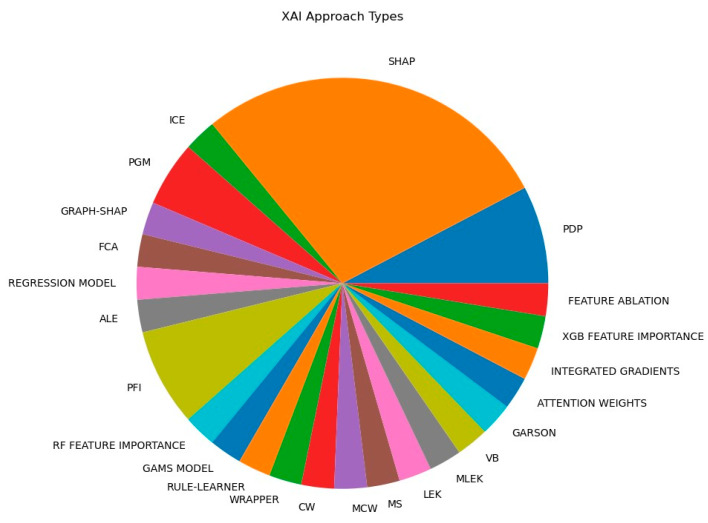
XAI approaches used in the epidemiological context, and frequency of use in the studies.

Figure 7 and Figure 8 depict the main ML and XAI approaches and their corresponding application frequencies within the selected epidemiological XAI research. A list of method abbreviations existing in Figure 7 and Figure 8 is presented in Abbreviations.

Figure 7 and Figure 8 visualize a dominance of XGB (using ML in the training step) and SHAP (using XAI in the interpretation step), as well as their joint application in the literature studied. Zopluglu argues that this results from XGB’s speed and performance based on parallelization, tree pruning, and hardware optimization [140]. Molnar argues for the first time that this may be grounded in the solid game-theoretical basis of SHAP [141]. At the same time, the higher proportion of the XGB- and SHAP-based pipelines (beside the prevalence of other tree-based ML and model-agnostic XAI techniques) indicates a lower presence of deep learning as well as the corresponding model-specific XAI techniques in the context of epidemiological AI studies of SARS-CoV-2. This is despite the fact that deep learning approaches (the corresponding XAI techniques) are a rapidly growing area of computer science.

Table 3 illustrates further modeling aspects employed in the different XAI-based epidemiological studies analyzed.

**Figure 9:**
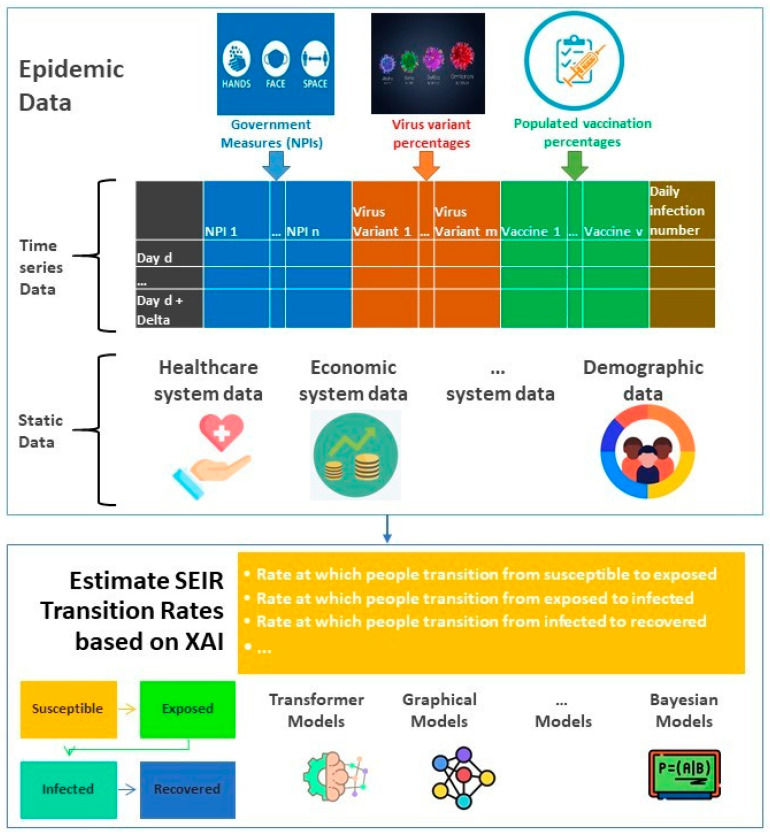
Hybrid SEIR-Deep learning approach in epidemiological pipelines.

SEIR-based ML models combine compartmental models with machine learning models to replace the fixed parameters of the former with time-varying parameters that are fitted using machine learning methods. Figure 9 illustrates the estimation of SEIR parameters based on AI-based approaches.

Time series data (Figure 9) includes the main epidemiological factors such as daily government non-pharmaceutical measures (NPIs), the percentages of different virus variants, as well as the proportion of vaccinated people. Static data include factors such as country-specific parameters of the healthcare system as well as its demographic and economic characteristics. The transition rates at which people move from one state of the SEIR model to another state can be calculated based on deep learning models fed with time series and static epidemic data. The deep learning models applied can be of different natures (e.g., pre-trained transformer-based, graphical, Bayesian, etc.).

Vega et al. [142] use a simplified probabilistic graph model (PGM) (e.g., probabilistic version of linear regression) to update the SEIR model parameters based on past information and estimated parameters in a previous iteration. Ref. [135] adopts a generalized additive model for each variable to be added to the SEIR model to represent the transmission rates.

A subset of studies is not designed to be used as real-time forecasting tools. Indeed, most of the studies employ fitted models to enhance the overall understanding regarding the effect of various influential features on pandemic progression, mortality rates, etc. Hence, they do not explicitly model the factor time. In contrast, a subset of studies (listed in the third column of Table 3) has explicitly modeled the factor time. These studies can be divided into two distinct categories: (a) Studies, which utilize dynamic time series models (i.e., RNN, LSTM, or CNN) to systematically incorporate the dependencies between consequent time points (listed in the fourth column in Table 3); and (b) studies, which treat each variable at each time point as a distinct input to the model (listed in the third column but not listed in the fourth column in Table 3). For example, Ref. [129] represents a combination of each NPI variable with how long the corresponding NPI has been in place as a distinct variable. Ref. [92] used, for each NPI, the lagged day with the largest Spearman correlation coefficient to generate the explanatory variables. Nonetheless, Ref. [127] uses a multi-stage (4-stage) LSTM, which, at each stage, forecasts a chosen target variable for one week ahead. The model elaborates on the initial first stage prediction to forecast an additional week, and it continues to implement this iterative approach, one stage at a time, to predict further into the future.

Graphical causal structure of the data is used in the studies presented in Table 3, not only for the sake of intrinsically interpretable model training [136,142], but also for incorporating prior knowledge into the resulting SHAP values [143]. In addition, creating associative rules as interpretable model-agnostic models is applied in [51,120].

The explanations provided by models in the sixth column of Table 3 are model-specific explanation types replicating an ML model’s internal mechanism. Such XAIs can better reflect the corresponding decision making of the models rather than explanations produced by model-agnostic methods. These XAI methods reflect:

- the internal connected neurons’ weights in [72];
- attention weights (to determine which input features should be given more attention over others, and the weight of importance for each historical temporal step) in [127]
- integrated gradient in [98];
- the XGB feature is important in [138], representing the percentage of trees that use a variable in the ensemble tree model.

Although the presented studies incorporate important data processing, training, and explanation tools, there is still room to reflect on enhancing the reviewed AI pipelines based on the existing scope of literature.

## 5. Discussion and Conclusions

**Figure 10:**
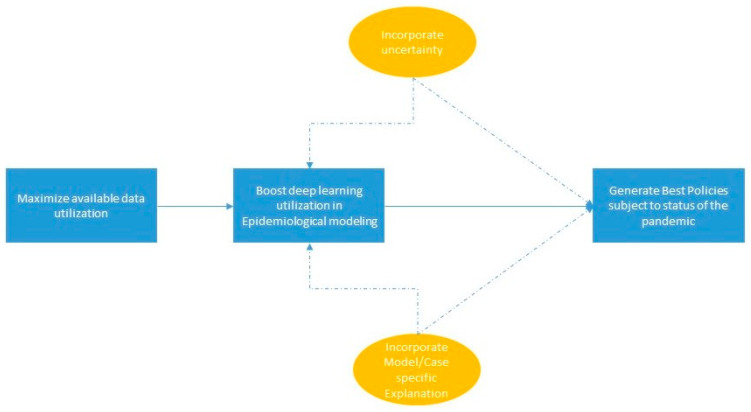
Main existing research needs in XAI-based epidemiological models of SARS-CoV-2.

The overall aim of this paper is to systematically figure out the main research gaps with regard to the methodological aspects of the epidemiological interpretable machine learning models of SARS-CoV-2. In Section 3, we developed a conceptual framework for the methodical pipeline of AI-based model development. The conceptual framework serves as a guideline for the analysis of epidemiological AI research in Section 4. Subsequently, we summarize the main existing research gaps in XAI-based epidemiological models of SARS-CoV-2 as data, modeling, explanation, uncertainty, and generation. Figure 10 visualizes the summarization of the main research needs related to each of the aforementioned research gaps.

More detailed research necessities are elaborated in the following subsections.

### 5.1. Data

Currently, the problem of missing, unbalanced, and sparse data is handled to a limited extent, especially by applying oversampling techniques such as SMOTE. SMOTE works based on the k-nearest neighbors’ principle and the principles of interpolation. However, such models can lead to the generation of poor, new, or unseen data. Upcoming epidemiological research needs to explore using more complex methods based on the variety of techniques mentioned in Section 3.1, especially generative approaches, e.g., AEs and GANs, to come up with a data scarcity issue.

In addition, while the datasets used in the recent epidemiological AI research are scattered and diverse, assessing the impact of different applied data sources in the analyzed literature in Section 4 on the corresponding results in a comparative way is crucial. A variety of influencing factors are used to generate results. Key data used in the reviewed epidemiological studies in our study are aggregated in Table 4.

Investigating the feasibility of integrating the above-mentioned data sources into comprehensive pipelines is a research need. This approach can lead to the creation of pre-trained epidemiological models based on the combination of existing large SARS-CoV-2 data sets. The resulting models can then be used for the next possible pandemic through transformer-based approaches based on the specific nature of new epidemic diseases or the corresponding specific spatial dimensions. The conceptual framework presented in Section 3 represents a starting point for tackling this research.

### 5.2. Modelling

Although the need to use deep learning models to address the spatial and temporal features of epidemics in epidemiological pipelines is urgent, we have found (in Section 4) that deep learning-based (as well as corresponding XAI) methods are not widely considered. The SEIR approaches can be enriched by using state-of-the art DL models. Currently, the time-dependent parameters of the applied SEIR models are often updated based on classic time series approaches or simplified probabilistic graph models [142]. Hybrid CNN-LSTM architectures as well as novel GNN approaches (presented in Section 3.4) are archetypal alternative approaches that can represent the time-varying character of SEIR model parameters in a robust and explainable manner.

The usage of CNN-based architecture approaches in the context of clinical studies is practiced [144]. However, the potential of CNN-based models has so far not been substantially exploited in XAI-based epidemiological studies. CNNs can be combined with the time-dependency-based architecture of existing LSTM models (mentioned in Section 4) to present multi-dimensional spatial-temporal representations of the pandemic. CNNs perform convolution operations in the upstream layers of the network, where the filters extract the most critical features to generate a feature map. The extracted features can not only be of spatial or temporal nature but also be used to recognize a range of government policies, including PIs and NPIs in a certain spatial context (cf. Figure 9). Further research is needed to explore these options.

In addition, other feature extraction methods mentioned in Section 3.2.2, such as knowledge embedding graphs, functional data analysis, and RFFs, have not been examined in epidemiological studies. By encoding the explanatory factors into dense and low-dimensional vectors, these methods can potentially boost the predictive power of AI models. Whether the inclusion of these methods enhances (or decreases) the explanation power of the models needs to be further studied. However, as mentioned in Section 3, through utilizing further AI and XAI techniques, it is possible to shed light on the features with the strongest impact on feature extraction methods. In addition, applying explainable graphical approaches to selecting the relevant features (noted in Section 3.2.3) has not yet been considered in epidemiological research.

Physics-informed neural networks (PINNs) [145] and neural ordinary differential equations (Neural ODEs) [146] are further noticeable modeling approaches that are not identified within the searched scope in Section 3 and Section 4, but need to be considered more in future XAI-based epidemiologic research. PINNs comprise neural networks that can add the SEIR models and the corresponding constraints as a regularization term in the loss function. The regularization term penalizes the training when SEIR models and the corresponding constraints are disturbed [147,148]. Neural ODEs combine the notions of ordinary differential equations (ODEs) and deep learning by parameterizing the derivative of the hidden state in the neural network. Given that the SEIR model can be expressed by an intractable system of ordinary differential equations, neural ODEs can devise a representing system that approximates the output of the model [149]. Future research shall demonstrate the added value of such approaches within future XAI-based epidemiologic research.

### 5.3. Explanation

The set of applied XAI methods shown in Section 4 is currently led by model-agnostic interpretation methods, especially SHAP-based approaches (see Figure 8 above). Other model-agnostic interpretation methods, i.e., surrogate meta-models introduced in Section 3.6.2, such as symbolic meta modeling, FCA, and anchors are not well known in the epidemiological context. In addition, while the high-performance degree of intrinsically interpretable approaches (especially the high performance of EBMs compared to non-intrinsically interpretable models) is indicated in the corresponding literature (cf. Table 2), such approaches are still not well known in the field of epidemiology. Moreover, the potential of model-specific interpretation methods (mentioned in Section 3.6.2) is, to date, not effectively utilized. Model-specific explanations can better replicate the ML models’ corresponding decision making than explanations created using model-agnostic methods. Model-specific CNN XAI approaches, such as gradient- and CAM-based methods, are proposed in papers belonging to the group of X-ray and CT imaging methods. The Grad-CAM interpretation method uses the gradients of the target class flowing into the final convolutional layer and, hence, can be used to produce visual explanations for any CNN-based model. It can be applied to the spatial-temporal epidemiological pipelines, too, indicating further research is needed in this area.

Case-based reasoning (CBR) techniques are another decent technique that can be adopted from the broad context of the literature in epidemiological XAI research. In the evidence-based medical domain, cases are the most specialized form of knowledge representation, consisting of both general understanding and human experiences, taking into consideration differences between the current case and typical or known exceptional cases [150]. Prototyping for the explanation of decision making within the training networks in the clinical and imaging-based studies of SARS-CoV-2 has been explored through CBR-based approaches [123,151,152]. However, this area of research has not yet been explored in an XAI-based epidemiological context.

Furthermore, comparing the explaining power and suitability of different XAI methods to epidemiological problems based on reliable criteria has not been elaborated in the existing literature. While some research, e.g., [153,154], has proposed guidelines for evaluating and scoring XAI methods from a human understanding point of view in medical image applications, neither a global evaluation schema nor specific evaluation metrics have been proposed as standard evaluation schemes so far. This needs to be further addressed in the medical AI literature as well as within the related epidemiological research. The relevant evaluation criteria must enable the epidemiological research to choose XAI methods, which not only efficiently convey the significance and magnitude of the effects of each pandemic explanatory factor in the spread of a pandemic but also efficiently explain the required time gaps, which could have been necessary to unfold the effect of each explanatory factor. This research can also possibly be extended to examine the fusion and hybridization of the existing XAI models.

### 5.4. Uncertainty

At the onset of pandemic phases, where the available data are scarce, not only the task of forecasting but also the task of explaining and generating suitable epidemiological policies are required to be inherently of a non-deterministic nature. The effect of data scarcity, both on model training and on model interpretation, is argued in Section 3.1 [19]. Scarce data necessitates the incorporation of uncertainty in the model training as well as in the model interpretation [17]. Incorporating uncertainty in model training and model interpretation level is practiced in computer vision literature. For example, [17] introduces ensemble models of pre-trained CNNs with large changes in CNN weights and applied uncertain-CAM approaches to their model explanation. In the epidemiological context, uncertainty is rarely performed based on Bayesian approaches [137]. Elaborating more on different non-deterministic approaches, including ensemble models (to best synthesize the predictions of multiple basic models) [93] and Bayesian neural networks (to infer distributions over the models’ weights and outputs) [155], can enhance future epidemiological pipelines.

### 5.5. Generation

The important role generative DL models can play within the scope of epidemiological studies is not elaborated on in the range of the reviewed SARS-CoV-2 literature studied in Section 4. Currently, most generative AI models are performed in the field of computer vision as well as in medical studies. A comprehensive study on the role of GANs in addressing the challenges related to SARS-CoV-2 data scarcity and diagnosis is presented in [156]. The use of GANs in the context of SARS-CoV-2 diagnosis is further studied in [38,157]. Generative epidemiological pipelines can create government policies along with different counter-factual scenarios as the pandemic spreads beyond the forecasting models. However, further research is needed to develop reliable and accurate results with GANs.

## Figures and Tables

**Table 1:** Overview and short explanation of dimensionality reduction algorithms used in SARS-CoV-2 literature context.

Method [Source]	Explanation
K-Means [50,51]	Clustering algorithms that can detect complex patterns based on a partition system to group data into several clusters
PCA—principal component analysis [26,51,52,53]	A statistical procedure, which relies on linear transformation for reducing the dimensionality of datasets while preserving crucial information
AE—auto encoders [40]	Perform dimensionality reduction similar to PCA. However, unlike PCA, which relies on linear transformation, AEs carry out non-linear transformation using deep neural networks
SOM—self-organizing maps [54,55]	Is an unsupervised machine learning technique to cluster the high-dimensional data into low-dimensional outputs consisting of a similar structure like artificial neural networks (ANNs), with the difference that the organizing maps in SOM use competitive learning whereas the ANNs use error correction learning such as back-propagation with gradient descent
LDA—Latent Dirichlet Allocation [56]	Is a Bayesian unsupervised clustering method that is often employed to cluster topics of a set of documents in each cluster
t-SNE—t-stochastic neighborhood embedding [57]	Is a kind of unsupervised non-linear embedding dimensionality reduction: It embeds the points from a higher dimension to a lower dimension trying to preserve the local structure of data
UMAP—uniform manifold approximation and projection [58,59]	Is a flexible non-linear dimension reduction algorithm based on Riemannian geometry and algebraic topology to learn the manifold structure of the data and find a low dimensional embedding that preserves the essential topological structure of that manifold
RFF—Random Fourier Features [60]	An approximate kernel method, which maps the given data to a low dimensional randomized feature space based on Euclidean inner product space

**Table 2:** Overview of intrinsically interpretable ML methods used in SARS-CoV-2 literature context.

Method [Source]	Description of Method
J48 [111]	Machine learning decision tree classification algorithm based on Iterative Dichotomies.
RIPPER [112,113]	Rule-based ML algorithm, in which rules are learned from the data directly.
pyFUME [112]	Can create rules based on fuzzy logic.
GAMs [114]	Used as non-linear regression tools that allow for non-parametric fittings of complex dependencies of responses.
GNAMs [115]	A hybrid ML-DL, which belongs to the GAMs family and learns a linear combination of multi-layer perceptron models.
EBM [116]	Explainable boosting machine is constructed with multiple hierarchically organized simple classifiers consisting of sequences of binary decisions and tree-based decision system.
JRip [109]	Rule-based classifier, which creates propositional rules that can be used to classify elements.
Quantum Lattice [25]	Inspired by the Richard Feynman path, which creates multiple possible graphical models composed of different mathematical operations. On selection of the best model, a Q-graph is created to provide the rationale behind a prediction. Further, a simplified equation for the model is obtained that provides insights into the mapping of inputs to outputs.
Bayesian networks [117]	Probabilistic graphical model for representing knowledge about an uncertain domain.

**Table 3:** Overview of modelling aspects in the XAI epidemiological studies.

Modelling Aspect	SEIR Based ML	Time Explicitness	Dynamic Time	Graphical Model	Rule Creating XAI	Model-Specific XAI
[Source]	[135,142]	[18,81,92,98,102,120,127,129,132,135,138,139,142]	[98,102,120,127,135,138,139,142]	[136,142,143]	[51,120]	[72,98,127,138]

**Table 4:** Overview of selected key data used in XAI-based epidemiological studies of SARS-CoV-2.

Paper	Selected Key Data
[133]	Compliance with NPIs, mobility patterns, work–life conflicts.
[134]	Income per capita, Population density.
[142]	Regional government policies.
[76]	Travel data, population density, medical endowments, environmental policy.
[143]	Socioeconomic disparities.
[120]	New cases, seasons, national lockdown, population vaccination number.
[102]	Population data, positive and total tests, number of cases and deaths, population vaccination number.
[81]	Population density, educational data, income data, household and housing data.
[129]	NPIs and how long which NPI has been in place.
[18]	Data on international travel bans, stringency of countries containment policies, Facebook users’ mobility data.
[130]	NPIs, different virus variants, average daily temperature, population characteristics, health expenditures, cultural participation data.
[135]	Confirmed case and deaths, Google mobility reports, government restrictions, demographic data.
[136]	Geographic data, e.g., proximity to major health facilities, churches, shopping centers and supermarkets, Average annual traffic density.
[131]	Health services indexes, GDP, behavioral risk factors.
[78]	Demographic data, economic data, health care data, unemployment, education, emissions.
[137]	Air pollution, proximity to industrial facilities, neighborhood and housing characteristics, age, poverty rate.
[61]	Dietary habits, past comorbidity prevalence, environmental policy factors such as seasonally averaged temperature geolocation, development indices.
[74]	Sex, age, ethnicity, comorbidities, socioeconomic data.
[92]	NPIs, influenza virology surveillance.
[132]	Vaccination data, wearing masks, mobility, government interventions.
[51]	Weather, culture, travel, health, economical data, development data.
[72]	Unemployment data, population density, air and rail transportation, urban population, gross national income per capita.
[127]	Demographic, public health data, population density, transportation, pollution, sex ratio.
[98]	Mobility, climate data, demographic data, virus variant frequencies.
[138]	Weather situation in the location of the infected person, medRxiv, and bioRxiv SARS-CoV-2 literature databases.
[139]	Mobility data, death number, patients in ICU, hospitalization by region.

## Supplementary Materials

The following supporting information can be downloaded at: https://www.mdpi.com/article/10.3390/life14070783/s1.

## Abbreviations

ML abbreviations	
ADAB	adaptive boosting model
ANN	artificial neural network
AR	autoregressive model
BI-LSTM	bidirectional long short-term memory network
BN	Bayesian network model
CATB	categorical boosting model
CBR	case-based reasoning
CONV-LSTM	convolutional long short term memory network
DT	decision tree
EBM	explainable boosting machine
ENR	elastic net regularization regression
GAMS	generalized additive model
GAN	generative network
GB	gradient boosting model
KNN	k-nearest neighbors model
LGB	light gradient boosting model
LOG-R	logistic regression
LR	linear regression
LSTM	long short-term memory network
MLP	multi-layer perception network
NN	neural network
ODEs	ordinary differential equations
PINNs	physics-informed neural networks
RF	random forest
RNN	recurrent neural network
SEIR	susceptible exposed infected recovered model
SVM	support vector machine
XGB	extreme gradient boosting model
XAI abbreviations	
ALE	accumulated local effects
CW	connection weights
FCA	formal concept analysis
ICE	individual conditional expectation
IG	integrated gradients
LIME	local interpretable model-agnostic explanation
MCW	modified connection weights
MS	most squares
SHAP	Shapley value-based explanation
PDP	partial dependence plot
PFI	permutation feature importance
PGM	probabilistic graphical model
VB	Variance-based model
